# Supplementary figures and images for: Metabolic modelling as a powerful tool to identify critical components of Pneumocystis growth medium
Source: PLoS Comput Biol. 2024 Oct 28;20(10):e1012545. doi: 10.1371/journal.pcbi.1012545 (PMC11542897; doi:10.1371/journal.pcbi.1012545)

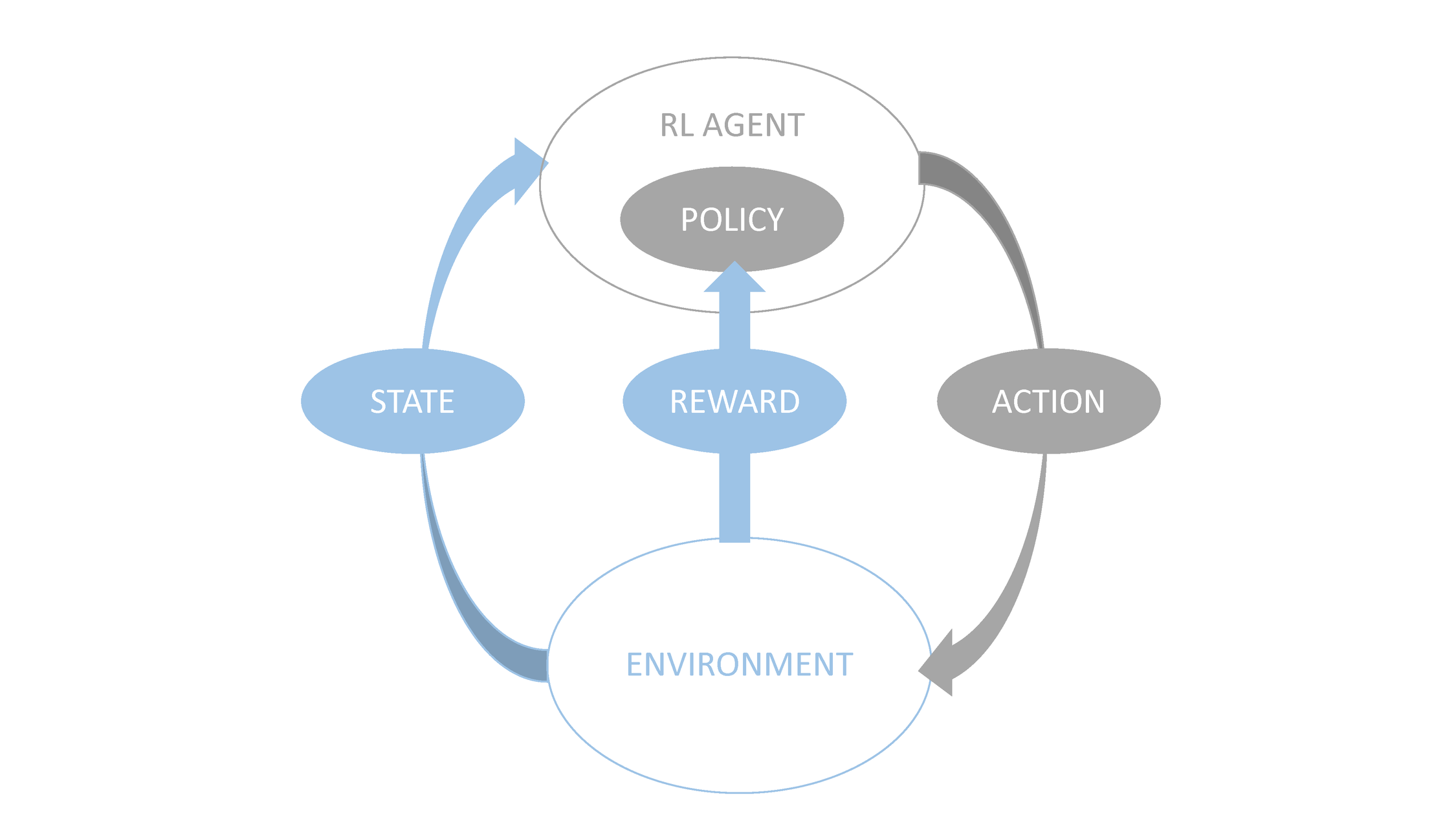

Supplement: S1 Fig — RL algorithms operate on states, actions, and rewards within an environment. The RL agent decides of which action to take based on the current state. The policy maps states to actions and is updated according to the obtained reward. The objective is to maximise cumulative reward by balancing exploration and high-reward actions. (TIF) [file pcbi.1012545.s008.tif]

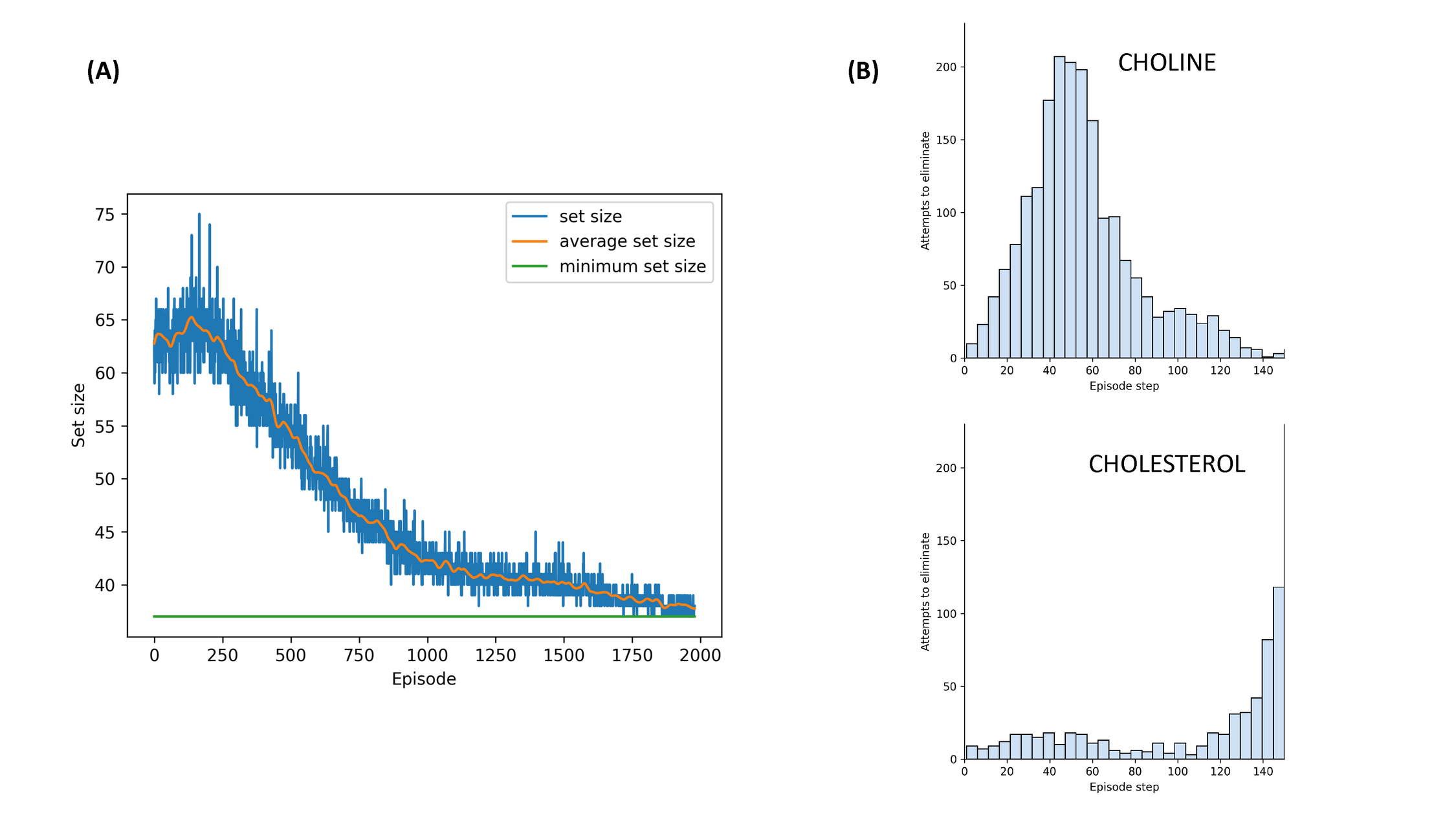

Supplement: S2 Fig — (A) The learning process of identifying and eliminating non-essential nutrients from the initial list of 155 nutrients is depicted by the blue and orange curves, which represent the precise and average sizes of the nutrient set after 118 attempts to remove nutrients by episodes. The value 118 represents the minimum number of nutrient deletions necessary to reduce the initial set of 155 nutrients to a minimal set consisting of 37 nutrients. (B) The distribution of the steps on which attempts made to eliminate choline, a non-essential nutrient (depicted on the top panel), and cholesterol, an essential nutrient (depicted on the bottom panel), from the initial list of 155 nutrients in Merali’s medium during each episode. The figures indicate that during the learning process the essentiality of cholesterol has been established, suggesting that it should not be removed at the onset of the episode. On the other hand, since choline is non-essential, it is advisable to eliminate it during the middle phase of the episode. (TIF) [file pcbi.1012545.s009.tif]
