# Supplementary material for: Metabolic modelling as a powerful tool to identify critical components of Pneumocystis growth medium
Source: PLoS Comput Biol. 2024 Oct 28;20(10):e1012545. doi: 10.1371/journal.pcbi.1012545 (PMC11542897; doi:10.1371/journal.pcbi.1012545)
Supplement: Attachment — Submitted filename: Response to Reviewer Comments.docx [file pcbi.1012545.s010.docx]

**RESPONSE TO REVIEWS - PCOMPBIOL-D-24-00648**

We are grateful to the Reviewers for the care, and effort they expended on our work and appreciate their helpful comments and suggestions. Our responses are detailed below.

**REVIEWER #1**

The manuscript "METABOLIC MODELLING AS A POWERFUL TOOL TO IDENTIFY CRITICAL COMPONENTS OF PNEUMOCYSTIS GROWTH MEDIUM" by Nev and colleagues presents the reconstruction of a genome-scale metabolic model of the fungal pathogen *Pneumocystis jirovecii*.

We respectfully point out that we developed a genome-scale metabolic model of the mouse-specific pathogen *Pneumocystis murina,* not the human pathogen *P. jirovecii*. This is now clarified in the text (as mentioned in our response to Reviewer #3, below).

In general, the reconstruction of metabolic networks in fungi remains a major challenge for systems biology and more efforts such as the present study are needed.

However, I do have a few critical points about the manuscript, which mainly refer to data availability, quality control of the genome-scale model, and results interpretation. Unfortunately, in the present manuscript version it is partly not comprehensible how the authors came to certain statements (e.g. about amino acid auxotrophies), because the corresponding results are not shown in the manuscript.

Introduction, 3rd paragraph: "we remain remarkably ignorant about the biology ... of this pathogen" is a harsh expression (also in the abstract). I am sure that researchers that study Pneumocystis jirovecii would disagree because the organism has been studied before using culture-independent methods, which also provide insights into the biology of the pathogen.

We have made the recommended changes (see lines 23-24 and 88-89 in the manuscript).

The abstract and introduction build on the central statement that P. jirovecii is not culturable. Yet, the last paragraph of the introduction cites a paper by Merali et al., where the organism was growth under laboratory conditions for a limited duration of time. Since this particular paper seems crucial in the context of the present manuscript, this paper needs to be introduced in greater detail already in the introduction. This should address the questions: What is exactly meant by "limited duration"? What was the innovation in the study that supported P. j. growth? What remains unclear after the Merali study and why is genome-scale modelling the method of choice to address open questions?

Merali *et al* reported that continuous axenic culture of *P. carinii* (a rat-specific pathogen) was achieved using a culture medium based on Minimal Essential Medium with Earle’s salts, supplemented with S-adenosyl-L-methionine, putrescine, ferric pyrophosphate, N-acetyl glucosamine, p-aminobenzoic acid, L-cysteine, L-glutamine, and horse serum [PMID: 10051654]. However, as we now state in the Introduction (lines 91-93), researchers worldwide have reported mixed success in replicating this culture system [PMID: 29895638; PMID: 34093506]. Therefore, the lack of well-established *in vitro* culture methods remains a major obstacle in *Pneumocystis* research.

Simple supplementation of growth media has not resolved this growth issue. It seems that nutrient supplementation alone is not sufficient to sustain growth. Instead, the organism must be able to maintain intracellular energetic and redox balance, for example. Therefore, to address this, we used metabolic modelling as an alternative approach (lines 94-100). At the time we initiated this study, Merali’s culture medium seemed the best starting point (control) to test our model.

Also, in response to the Reviewer’s comment, we have deleted the words "limited duration" to avoid confusion.

Section METABOLIC MODEL DEVELOPMENT: "Pneumocystis [...] is incapable of synthesising all 20 essential amino acids". That seems very unlikely. For instance, there are plenty of different metabolic routes to produce alanine, aspartate, and glutamate, so that established auxotrophy prediction tools like "GapMind" do not make statements about auxotrophies for these amino acids. Even more importantly: It is unclear how auxotrophies were assessed or predicted. What precursors/pathways were considered to make the statement of the incapability to synthesise all 20 proteinogenic amino acids. Moreover, Figure 7A clearly shows that the model can produce biomass without certain amino acids in the growth medium, indicating that the organism is capable of synthesizing at least some amino acids.

We apologise for the confusion. For clarification, we have now amended the text to specify *“*synthesise *de novo*” *(*see lines 174-175). *Pneumocystis* species lack ~80% of the amino acid biosynthetic genes present in other yeasts [PMID: 26899007; PMID: 29899010], and hence *Pneumocystis* species are unable to synthesise any of the 20 amino acids *de novo.* However, as the Reviewer correctly infers, some amino acids may be generated by interconversion from others and/or through degradation of proteins or peptides. For instance, *Pneumocystis* can interconvert glutamine and glutamate as well as serine and glycine (Figure 7A).

Biomass reaction. It is mentioned that the biomass reaction was defined based on ""genome data, software tools, pre-existing metabolic models of species closely related to Pneumocystis, and the limited information about the pathogen’s composition available in the literature". However, the described procedure misses essential information to make the biomass reaction definition reproducible. For instance "the aid of a java application" is too vague and more details need to be provided (what is this application, what are underlying assumptions and data sources?). The same applies to this part: "majority of the content for the soluble pool of small metabolites was derived from a comprehensive general list". Please provide more details on this "general list".

As requested, we have added more details in the text for clarification (see lines 205-209 and 212-213).

There are no results on the technical quality of the reconstructed GEM. This could for instance be done using MEMOTE (<https://doi.org/10.1038/s41587-020-0446-y>).

We have added a subsection on technical quality of the reconstructed GEM in the Materials and Methods (*Technical quality of the model*, lines 709-725). The MEMOTE report is included in Supplementary Information (SI 7).

Optimization of Merali's medium. I can't find a table that represents the reconstructed Merali's medium, which is used as reference for several analysis presented in the manuscript.

The reconstructed Merali’s medium is present in Supplementary Information 3 (*List of transport reactions under Merali’s medium conditions*) and is discussed in the Materials and Methods (*Merali’s culture medium composition*).

The metabolic model should be provided in SBML format also already for the review process, since this is the main result of the study.

The developed model in SBML format is included in Supplementary Information (SI 6).

Please add line numbers. This makes reviewing easier.

We have added line numbers, as requested.

**REVIEWER #2**

Nev and colleagues use metabolic modelling to identify necessary components of the fungal pathogen Pneumocystis growth medium. P. jirovecii is an important pathogen that cause lethal pneumonia in immunocompromised patients. The fungus is difficult to study due to a lack of long-term culture method. Here, the authors leverage on available genomic data, insights from more than 30 years of culture attempts to model the metabolic fluxes in P. murina, the sister species of P. jirovecii.

This study addresses a need in the research community. The manuscript is well written, the methods are generally appropriate, and the conclusions are supported by the results provided. The comments below are provided to discuss some concepts and potentially improve the manuscript.

This study is a proof of concept showing that metabolic modelling can help achieving a reliable continuous culture of Pneumocystis organisms in an axenic setting. One of the main predictions of the model is Pneumocystis reliance on glucose. This is hardly new and has already been inferred from Pneumocystis gene catalogs (See Ma et al. 2016 for example). It is my understanding that the authors took the safe route and showed only predictions that fit the current narrative. It should be noted that the link between gene loss and metabolic dependency is far from being proven. As is the model seeming under-utilized. Can the authors present or emphasize on new or counterintuitive results not predicted in the literature?

I understand that answering this question can be risky because this paper is a purely in silico study, and producing predictions without experimental backup can be hard to defend. However, it would be of interest to to Plos Comp Biol readers.

We agree that the choice of glucose as carbon source was intuitively obvious! We selected the glucose-only condition as a negative control because it lacks other nutrients likely to be essential for *Pneumocystis* growth as mentioned in the section on *Model validation*. We then discussed model’s predictions in subsequent sections (see *Model predictions* and *Model predictions and empirical data*). We would argue that some of these predictions were not intuitively obvious. For example, the critical requirement for lipids for *Pneumocystis* and the relative importance of amino acids, fatty acids, and steroids for *Pneumocystis* growth. We also employed the model to test whether *Pneumocystis* is a SAM auxotroph because differing opinions on this have been reported (please see lines 453-458). We believe that these results are novel but, as suggested by Reviewer, they do require testing experimentally.

The reliance on Merali et al paper to confirm their predictions is a major - but easy to fix - drawback. The method described in this paper does not work (even mentioned by the authors). Efforts to replicate this study have failed. Melanie Cushion et al (ref #16) has nicely summarized efforts done in supplementing the media. Therefore, the reliance of Merali is not warranted.

With respect, our goal was to build on, not rely on the findings of Merali and co-workers. As mentioned above, we used Merali’s growth medium as a starting point for our study [PMID: 10051654], which we initiated well before Cushion’s excellent review was published [PMID: 34093506].

The other argument is Pneumocystis is an obligate biotroph that apparently need a host. With that in mind, axenic methods are unlikely to work and co-culture methods are more successful (PMID: 38117035). This aspect is not captured by this modelling. The effect is exacerbated when an inappropriate dataset is used such as Merali. Can the authors please comment>

We respectfully point out that the paper the Reviewer cites describes attempts to grow *Pneumocystis* on precision cut lung slices (PCLS) rather than the co-culture of fungal and mammalian cells. (We wrote a comment on this paper [PMID: 38345378].) Even using this PCLS model, Jay Kolls’ team was only able to report longer maintenance of *Pneumocystis* cells, not actual growth [PMID: 38117035]. Also, for the record, we viewed Merali’s dataset as a starting point, not as “inappropriate”. (Please see our response to the Reviewer’s previous point.)

Nevertheless, the Reviewer makes an excellent point. Co-culture with mammalian cell lines is more likely to yield *Pneumocystis* growth, albeit *ex vivo,* and hence we discussed taking this approach many times. However, our overall objective was to identify media that support axenic growth. This is because, although risky, axenic growth would immensely empower the dissection of *Pneumocystis* pathobiology by facilitating forward genetic screens, reverse genetics, molecular analyses, structural studies, etc.

From the modelling perspective, it would be interesting to integrate metabolic models for *Pneumocystis* species with their specific mammalian hosts. Indeed, we mentioned this in the Discussion (lines 551-553). Nevertheless, to address the Reviewer’s comment, we have strengthened the point that the integration of metabolic models could enhance the understanding of the metabolic relationships between pathogen and host (lines 553-556).

Finally, adding elements to growth media is not enough. And many of these elements have been tested already (see ref # 16).

We agree with Reviewer. As we state above, our prediction was that the supplementation of predicted auxotrophic requirements would be insufficient to achieve axenic growth of *Pneumocystis.* For this reason, we felt that our modelling approach potentially provides a powerful tool to address this key issue in *Pneumocystis* research. In response to the Reviewer’s comment, we have now strengthened this point in the text (lines 96-100 and 494-497).

As mentioned above, we started our modelling before Cushion published her review (which we quote numerous times in our paper: PMID: 34093506).

Specific comment

In particular, we estimated Pneumocystis protein and DNA content from genomic data with

the aid of a java application [37].

What the tool is doing exactly?

We have added more details in the text for clarification (see lines 205-209).

“FBA assumes that the metabolic system is in a quasi-steady state, where metabolite

production and consumption are balanced within the cell.”

Can public RNA-Seq data be used to initialize the model? For example, gene expression can be used to capture the cellular state in different conditions (e.g. ref 32). Where are the codes (or GitHub repo)?

Indeed, public RNA-Seq data can be used to contextualise the model. The model’s initialization, however, can be done by using taxonomy, EC numbers, and genomic data in the Constellab platform.

The code used for the model’s reconstruction and FBA can be found in the following GitHub repository (version 0.6.3 was used in this study, tag 6.3 in the code): <https://github.com/Constellab/gws_gena> (lines 909-911)

The code used for the RL approach can be found in the following GitHub repository:

<https://github.com/nevolga/optmed> (lines 912-913)

Note that a digital lab is to be created either on premises or in a cloud to run the code. The guidance can be found here:

<https://constellab.community/bricks/gws_academy/latest/doc/getting-started/b38e4929-2e4f-469c-b47b-f9921a3d4c74> (lines 914-917)

We have also included Data availability statement in the text (lines 907-917).

“To ensure that the model is free of technical errors, the model is

usually tested against known constraints. To test whether the model accurately represents the metabolism of the organism, the predictions of the model are compared with experimental data obtained under various environmental conditions.”

This sentence is unclear. There are no environmental conditions available. Also, culture system does not recapitulate in vivo conditions.

We apologise for this confusion. Here, we discuss in general how metabolic models are validated and tested under 'normal' conditions where environmental data are available. We have modified the text to clarify this (lines 273-274). Unfortunately, this is not the case for *Pneumocystis*.

“data are limited and often obtained under ex vivo conditions (i.e. growth with cultured mammalian cells) which are not suitable for the model’s validation. To validate our metabolic model, we employed in vitro growth culture medium conditions designed by Merali et al. [30]”

As discussed, Merali et al work is not appropriate.

As mentioned above, we do not view Merali’s work as inappropriate, but as a useful starting point for our work.

“conditions and discovered 91 zero fluxes associated with extracellular transport reactions (SI 4, Table S5). These fluxes do not appear to contribute to biomass production and, therefore, the corresponding ambient nutrients from the medium can be excluded. We compared the nutrients identified as non-essential for growth by the RL approach with nutrients whose corresponding flux values were zero. The RL algorithm identified a significantly greater number of non-essential nutrients, considerably simplifying the original 155-component list of Merali’s medium composition to only 37 nutrients.”

This whole paragraph is about predicting non-essential genes. Can RL be trained on RNA-seq data? Or what would be the outcomes if RL is trained on different datasets?

To clarify, this paragraph is not about non-essential genes, but about non-essential nutrients (i.e. nutrients that are not required for growth).

As we have shown using Merali’s case study, the RL approach is a powerful tool for optimizing growth medium composition and can be trained similarly on other nutrient datasets.

“Merali et al did not experimentally detect any activity of the SAM synthase and thus

suggested that P. carinii is a SAM auxotroph … suggest that Pneumocystis may not depend on its host for the supply of this important cofactor.”

This paragraph has an historical value and would be appropriate for a review, but here it is confusing. Pneumocystis organisms definitively have a SAM synthase. This reinforces my suspicion that Merali et al work is not reliable.

Our modelling indicated that SAM is not obligatorily required in the growth medium when methionine is present, suggesting that *Pneumocystis* is not a SAM auxotroph (as suggested by Merali *et al*. [PMID: 10051654])

“We have developed, for the first time, a genome-scale metabolic model for Pneumocystis. In”

Efforts to model metabolic pathways have been attempted before (for example Porollo 2014, PMID: 25202338; Hauser et al. PMID: 21188143)

The studies referenced by Reviewer, mapped certain *Pneumocystis* peptides and enzymes identified from genetic onto metabolic pathways for comparison with phylogenetically close fungi, such as *S. pombe*, *S. cerevisiae*, and *A. fumigatus*. This might be considered as a step towards the development of a metabolic model. However, these studies did not reconstruct metabolic networks. Neither did they utilise Flux Balance Analysis (FBA) to calculate metabolic fluxes through the *Pneumocystis* network. We believe that our model is the first *in silico* genome-scale metabolic model (GEM) of *Pneumocystis* growth and metabolism.

**REVIEWER #3**

The here presented manuscript presents a genome-scale metabolic reconstruction of Pneumocystis murina, a representative of the Pneumocystis genus.

The authors describe reconstruction details, FBA based simulations and model predictions. One major drawback of Pneumocystis research appears to be the inability to have it grown in vitro so far. Hence, a genome-scale metabolic model (GEM) to shed some light into its metabolic components features appears timely.

Although the limited knowledge about Pneumocystis biology hindered some parts of the reconstruction and FBA-based analysis, I have major concerns regarding the methodology and miss supplementary information with model and simulation details. Moreover, the predicted minimum growth medium was not experimentally tested and thus remains hypothetical.

Major points

--

In the model predictions section, how was influx modeled? Were all influx exchange-reaction-related bounds open or in any way constrained to specific flux values e.g. as given by the definition of the Merali medium? This has definitely impact on model predictions and needs to be stated here.

We reasoned that the inability to achieve axenic growth of the fungus could be due in part to its dependence upon the host to repair metabolic imbalances (lines 27-28, 96-99 and 494-497). Therefore, with a view to permitting the potential excretion of metabolic byproducts, in the absence of empirical data we opted not to constrain external fluxes (influxes according to Reviewer). We have now added the corresponding information on upper and lower bounds for the transport reactions (external/exchange reactions from the reconstructed Merali’s medium) in Supplementary Information 3.

Also, a complete Suppl. table with model predictions (and used influx values and achieved FBA objective function values as well as all model flux values) is missing.

As requested, we have now added Supplementary Tables S9 (A)-(D) (see Supplementary Information 5) with model predictions used in this study. We have also included all model flux values under the most important conditions. (For practical reasons we did not include all conditions (see Supplementary Information 5, Tables S10-S12)).

- Moreover, since Arginine biosysythesis is not complete as shown in Fig 2B, how do the authors explain that missing arginine in the medium has such low impact? It does not matter, for predicted viability (FBA>0) to which extend an amino acid (or any other factor) contributes to the biomass equation. If it is essential, FBA is necessarily 0. Obviously the individual lack of each amino acid could be compensated as shown in Fig. 6. How, is not explained. The authors need to address how the missing amino acids are produced by investigating the respective FBA fluxes or even better by running reaction essentiality analysis with vs. without a given amino acid using e.g. FVA.

There appears to have been some confusion. We did not use classical FBA. Rather, we relaxed the quasi-steady state assumption (QSSA) to obtain an optimal solution for the *Pneumocystis* metabolic network (because this network has major gaps and because of the lack of experimental data). We have modified the text to make this more clear (lines 698-707).

Experimental measurements and model parameters (such as flux capacities) are typically subject to uncertainties. One way to cope with this biological uncertainty is to relax the QSSA by assuming that certain intermediate metabolites may not reach steady state, reducing the number of constraints and equations. This enables the model to maintain flux to biomass production by adjusting other parts of the network to compensate for missing components. Consequently, the model focuses on overall flux balance rather than precise stoichiometry, allowing for non-zero biomass flux even if certain biomass precursors are missing. QSSA relaxation approach has been successfully applied to a range of GEMs [PMID: 28325918; PMID: 31510676; PMID: 37697651; Zavlanos MM, Julius AA. Robust flux balance analysis of metabolic networks. Proceedings of the 2011 American Control Conference. 2011. p. 2915–20], notably in the work of Reznik et al [PMID: 24009492] which demonstrated that flux imbalances could provide valuable information about in vivo metabolite concentrations.

The section on glycine/serine and SHMT is unclear. A gene KO has no effect, but if the reaction is knocked out, the authors report an effect (no serine-glycine interconversion). The authors need to explain this, e.g. has the respective gene-to-reaction rule an isozyme taking over the role for enabling the reaction? Otherwise it is not clear how the reaction was functioning after the gene-KO. The effect should have been the same as seen by the predicted flux after reaction-KO.

We believe this is a misunderstanding. The model predicts that omitting either serine or glycine from the medium does not have effect on the biomass flux, and omitting both amino acids from the medium has little effect. In contrast, omitting both amino acids *and* knocking out SHMT significantly decreases the biomass flux (Figure 7A). We did not mean to imply that the reaction was functioning after the gene knock-out. We have revised the text in an attempt to make this more clear (lines 333-334).

Also, I miss gene-to-reaction rules in the supplements for each reaction and the evidences on which basis reactions were added to the final model. Comprehensive information which reaction associates to which metabolic pathway should also be given.

To address this, we have now added the required information to the Supplementary Information 2, Tables S4-S5.

When investigating the role of lipids the authors again need to explain how influx was modeled (constrained or not to certain values). Also again, if any component is part of the biomass composition, its prohibitited influx should result in FBA=0, unless the compound is produced elsewhere.

Please refer to the explanation above describing our use of the relaxed QSSA.

The paper would benefit if the authors would use their model to predict the reactions necessary to produce certain sterols and fatty acids, which were added to the biomass reaction based on information in references 38-40, but are not part of the growth medium by Merali. This can only mean the model is able to synthesize these, which is not necessary if additionally given in the medium - as has been simulated and shown in Fig. 5. On its own this is a trivial result. But if the authors add predictions/simulations on which pathways are involved in the biosynthesis and e.g. what energy demands are circumvented by supplementing the respective metabolites to the medium, the advantage of having the model at hand would be demonstrated.

Again, we believe this is a misunderstanding. *Pneumocystis* lacks metabolic pathways required for *de novo* synthesis of fatty acids and sterols included in the biomass reaction. As we stated in the manuscript (lines 349-352), *Pneumocystis* has been shown to assimilate these from the medium. This is included in our model (see Table S1 and KEGG maps *Fatty acid biosynthesis* and *Steroid biosynthesis*).

Concerning the growth medium optimisation, it is certainly interesting to see this great reduction in necessary metabolites from >800 to 37. Toolboxes like CobraPy allow to predict a minimal medium, which could be exploited to find all possible variants with a minimal number of metabolites. How does the presented RL based approach compare to this rather straight forward approach included in CobraPy? Are the core nutrients found also in there?

CobraPy employs a standard FBA approach that assumes that the QSSA is met. As stated above, we had to relax this assumption due to the complexity of our model. Therefore, it was not possible to compare our RL approach with the one included in CobraPy.

Table S5 presents 91 zero fluxes of given medium components. Does this imply these are not taken up by Pneumocystis at all or are these values associated to the minimum already?

Yes, this is correct. We assume that zero fluxes imply that *Pneumocystis* does not take up the corresponding nutrients from the medium. For clarification, this is now stated as a footnote in Table S7 (previously S5).

How do the authors explain that e.g. L-argininium(1+) is a core metabolite after RL application according to S6, but the respective flux in S5 is 0?

We believe this is a misunderstanding. In Table S7 (previously S5) this has a non-zero flux, which is consistent with its core metabolite status in Table S8 (previously S6).

How should negative and positive fluxes in S5 be interpreted, since in this table they reflect the Merali medium, for which there should be only uptake or zero flux?

Positive fluxes imply that *Pneumocystis* takes up the corresponding nutrients from the medium, whereas zero fluxes imply that *Pneumocystis* does not take up the corresponding nutrients from the medium. Since we opted not to constrain external fluxes in the absence of empirical data (with a view to permitting the potential excretion of metabolic byproducts), some fluxes may have negative values.

How does metabolomics data (if avialable) before and after Pneumocystis growth compare to these presented values?

Unfortunately, no metabolomics data are available.

In the methods section, the quadratic objective instead of regular FBA is insufficiently explained and justified. Why has the presented formular been adapted instead of regular FBA? Why have relaxation terms been added to the problem formulation? Has this been used and published elsewhere?

Please refer to the explanation above describing our use of a relaxed QSSA.

What does "see FBA analysis" refer to as there is no such section?

We apologise for this mistake. The wording has now been corrected to “see *Flux Balance Analysis*” (line 696).

The model should be tested with MEMOTE and should be available in SBML format.

We have added a subsection on technical quality of the reconstructed GEM in the Materials and Methods (*Technical quality of the model*, lines 709-725). The MEMOTE report is included in Supplementary Information (SI 7). The developed model in SBML format is included in Supplementary Information (SI 6).

Minor points

--

Introduction misses a description of existing fungal genome-scale metabolic models.

To address this, we have added a brief overview of existing fungal GEMs in the Introduction, as requested (lines 125-128).

Table 1 is potentially interesting for follow-up studies. It misses the involved lost or retained metabolic reactions, however, and I expect these to see in a Supplementary Table.

To address this, we have included a comparison of the list of EC numbers from the yeast consensus model [PMID: 31395883] versus EC numbers present and lost in the *P. murina* model developed in this study (Supplementary Information 2, Table S6).

In addition, we have created metabolic maps using the KEGG Mapping Tool to compare the *P. murina* metabolic network with a network that combines reactions in both of the model yeasts *S. pombe* and *S. cerevisiae*. Metabolic pathways present in *P. murina* are highlighted in blue, whereas those pathways present in these model yeasts, but not in *P. murina*, are featured in red. We believe that this way it will be clearer for the Reader to see the gaps in the *Pneumocystis* metabolism.

These KEGG maps can be found here: <https://figshare.com/s/3a260486d5d8a6067b16>

We have also included Data availability statement in the text (lines 907-917).

The section "Biomass equation" would benefit from a reference to pmid20430689 for further information on biomass objective function.

This reference has now been added, as suggested (see line 186).

The first paragraph on FBA lists some FBA application papers, the youngest being published in 2012. Please add 1-3 more recent applications.

More recent references have been added, as suggested (see lines 227-232).

The authors repeat the understandable limitation of Pneumocystis not growing in culture and therefore data for validation is scarce. Nevertheless, the model validation section needs more explanation, mainly to describe what the authors did (or not) to remedy the discrepancy between observed and simulated growth.

As mentioned above and in the manuscript, minimal experimental data on the axenic growth of *Pneumocystis* are available, and those that are available are questionable. To minimise potential discrepancies in this *in silico* study, we performed extensive (and extremely time-consuming) manual inspections on apparent gaps in the metabolic network generated by the bioinformatic analyses (see lines 156-161). This included protein-by-protein analyses of structural domains. Gaps were also inspected by examining *in vivo* RNA sequencing datasets kindly provided by Jay Kolls (line 159).

Next to confirming observations by Merali and others which metabolites appear important, did the employed approach also predict essential nutrients that so far were not mentioned in the literature?

Yes! The general metabolic requirements of *Pneumocystis* were identified during the construction of the metabolic model. This revealed essential nutrients that had not been considered previously, most notably specific lipids, e.g. sitosterol, elaidic acid, pentadecanoic acid, henicosanoic acid (see Supplementary Information 5, Table S12). This study is a proof of concept showing that metabolic modelling could help to design reliable continuous culture media for *Pneumocystis* species.

The discussion mentions for the first time clearly that a GEM model of P. murina was reconstructed. How different or similar is this related to e.g. P. jirovecii? How much are genome annotations differing, which potential metabolically relevant genes are present in one, several or all? What about homology-searches of the organism-specific Pneumocystis species against e.g. S. cerevisiae genome annotation and comparison against the Yeast consensus model, which is one of the best curated ones? The understandable reasons why to reconstruct a P. murina model as starting point and to resemble the Pneumocystis genus should be made clear in the very beginning of the results. Also, throughout the paper, instead of describing "metabolic model for Pneumocystis" or "critical components of the Pneumocystis growth", etc. it should be made clear that either P. murina or the genus is meant.

We have now clarified this, as requested. The focus on *P. murina* is now mentioned in the Introduction, where we also point out that minimal metabolic differences exist between this species and its cousins *P. jirovecii* and *P. carinii* [PMID: 26899007] (see lines 104-108). The Reader is now reminded of this focus at numerous points during the manuscript (for example, on lines 171, 543, 574, 580, etc.).

In the materials and methods section, the description "Finally we checked the obtained list of enzymes against the biochemical reaction database BRENDA [34] and RNA Seq data [32], and also used available, albeit limited, experimental data on Pneumocystis to curate enzymes manually." appears not precise enough. How was this done. The supplements with the affected reactions should be referenced here.

We have included the list of obtained enzymes together with the corresponding *P. murina* genes and evidence in Supplementary Information 2, Table S5.

Are the gene-to-reaction rules derived by the ConstellabTM software tool different or equal for the same reactions present also in the yeast consensus model (https://github.com/SysBioChalmers/yeast-GEM)? A comparison would improve trust in the given model, which is otherwise based on proprietary black-box software.

A direct comparison of Constellab outputs with those of the yeast model is challenging because there are differences in their metabolite nomenclatures. Also, we compared *P. murina* to a composite metabolic network that includes both *S. pombe* and *S. cerevisiae* reactions (lines 805-806) because *P. murina* is more closely related to *S. pombe*. Therefore, to address this point we now include a new Supplementary Table that compares the enzyme EC numbers in the yeast consensus model with those in our *P. murina* model (Supplementary Information 2, Table S6).

The deleted "isolate" reactions may not be relevant for growth, but this largely depends on the given growth medium. As the authors repeatedly state, stable in vitro growth so far is not possible and only the Merali medium supports growth for some time. This, however, does not rule out other possible growth media compositions, for which one or several of the deleted reactions may become relevant. Even if not, these may be important for pathogenicity and thus, helpful to other researchers and the community. At the minimum, their reaction details should be added and described in the supplements, e.g. in SI2.

This appears to be a misunderstanding. The isolate reactions were present in Supplementary Information 2, Table S3.

The gap filling procedures need to be elaborated. Is this step included in the pipeline by the Constellab software? Since viability of the model was achieved, the question arises, which and how many gaps were filled given which resource. Such information should be in the per reaction supplementary tables, e.g. in table S4.

Yes, the gap filling procedure is included in the pipeline in the Constellab software. To automatically fill “metabolic gaps” in the network, we exclusively relied on the *Pneumocystis* taxonomy (4753) to avoid filling “actual metabolic gaps” that reflect the pathogen's dependence on its host, as this may potentially lead to inaccurate predictions. As a result, no new reactions have been added.

In the methods description of Merali’s culture medium composition, the culture medium appears to be based on P. carinii, however, the model built here was P. murina. The text should reflect again on the similarity (or not) of these strains.

As mentioned in response to a previous point, we now mention in the text that minimal metabolic differences exist between *P. murina, P. jirovecii* and *P. carinii* [PMID: 26899007] (see lines 104-108). Therefore, our rationale for focussing on *P. murina* was as follows. Inevitably, animal experiments will be required to follow up on observations generated *in vitro* using axenic culture (for example, to confirm the pathogenicity of *Pneumocystis* cells grown *in vitro*). Mice are less expensive than rats, and recent advances in mouse transgenesis have been impressive. Therefore, with a view to opening our potential findings to as broad a scientific community as possible, we built the model for the mouse pathogen (*P. murina*). Any subtle metabolic differences between *P. murina, P. carinii* and *P. jirovecii* can be addressed at a later date simply by adapting our metabolic model.
