# Supplementary material for: Metabolic modelling as a powerful tool to identify critical components of Pneumocystis growth medium
Source: PLoS Comput Biol. 2024 Oct 28;20(10):e1012545. doi: 10.1371/journal.pcbi.1012545 (PMC11542897; doi:10.1371/journal.pcbi.1012545)
Supplement: Attachment — Submitted filename: Response to Reviewer Comments.docx [file pcbi.1012545.s011.docx]

**RESPONSE TO REVIEWS – PCOMPBIOL-D-24-00648R1**

We are grateful to the Reviewers for the additional comments and suggestions. Our responses are detailed below.

**REVIEWER #1**

The revised manuscript demonstrates several improvements, including the inclusion of the metabolic model in SBML format. However, two of my main concerns from the previous report remain unresolved:

Auxotrophies: In my previous report, I raised concerns regarding the predictions of amino acid auxotrophy. While the authors have expanded the discussion on amino acid auxotrophies, I remain unconvinced by the current response. The manuscript and response letter still assert that *Pneumocystis jirovecii* is unable to produce any of the 20 proteinogenic amino acids. This assertion contradicts the results presented in the section "In silico Drop-off Experiments" and in Figure 6. In this section, the manuscript describes the results as follows:

"The results suggest that not all amino acids contribute equally to Pneumocystis growth. In particular, the model predicts that a deficiency of isoleucine or histidine in the medium results in a significant reduction in biomass flux, whereas arginine or tyrosine deficiencies do not (Fig 6)."

If the organism is indeed auxotrophic for all 20 amino acids, the maximum flux through the biomass reaction should be reduced to zero when the organism is unable to synthesize the respective amino acids. However, Figure 6 clearly shows that the relative biomass flux remains greater than zero for all amino acids tested through exclusion from the in-silico growth medium. This inconsistency needs to be addressed to clarify the organism's metabolic capabilities.

Herein lie two issues: ***Auxotrophies*** and ***Zero Flux Values***.

***Auxotrophies*:** As we understand it, the Reviewer is concerned about an apparent contradiction between the inability to synthesize any of the 20 standard amino acids *de novo* [PMID: 26899007; PMID: 29899010] and the observation that *Pneumocystis* is not auxotrophic for all 20 amino acids.

With respect, we would like to point out that: (a) we do not say that *Pneumocystis* is auxotrophic for all 20 amino acids; and (b) the sentence highlighted by the Reviewer (lines 303-306) relates to a different discussion about the fact that amino acids do contribute differentially to biomass based largely on their differential usage in protein synthesis. In our previous submission, this was in the subsection entitled *In silico drop off experiments*. Then, in the next subsection (*In silico gene knockout experiments*), we went on to discuss how metabolic interconversions between certain specific amino acids (glycine and serine, for example) allow the presence of one of these amino acids to essentially suppress an auxotrophic requirement for the other, and *vice versa* (now in lines 317-321). This addresses the Reviewer’s concern about a lack of *de novo* synthesis *versus* auxotrophic requirements. Nevertheless, we now realise that, by splitting these paragraphs into different subsections, we broke the logical progression from the differential biomass contributions of amino acids to their metabolic interconversions, both of which influence outcomes. Therefore, to address this Reviewer’s concern, we have repaired this logical progression by placing these paragraphs together in the same subsection, now entitled *In silico drop off and gene knockout experiments* (please see lines 300-334).

***Zero flux values*:** The Reviewer is also concerned about the lack of zero flux values. Some confusion remains around this point. If classical Flux Balance Analysis (FBA) had been used to calculate the metabolic fluxes, then the Reviewer would be correct to suggest that the removal of an essential amino acid would result in a predicted biomass flux value of zero. However, as stated, we did not use classical FBA in this study. Instead, we relaxed the quasi-steady state assumption (QSSA) to obtain an optimal solution for the *Pneumocystis* metabolic network. Therefore, predicted biomass flux values of zero were *not* expected. As we stated, this relaxed QSSA approach was necessary because of the significant gaps in the network and the limited availability of experimental data. We described our approach in the manuscript (lines 693-702).

Biomass Reaction: Although the authors have added further details on how the biomass reaction was inferred, the information provided is still incomplete, which hinders the reproducibility of the biomass formulation in the metabolic model. Specifically, the revised manuscript states:

"In particular, we estimated Pneumocystis protein and DNA content from genomic data with the aid of a biomass tool developed by Santos & Rocha [49]. This tool calculates the microbial biomass composition in amino acids and nucleotides based on genomic and transcriptomic data, accepting input files in FASTA format and transcriptomic data in CSV format. It is reported to serve as a viable alternative in the absence of experimental data [49-51]"

To ensure reproducibility, it is essential to provide the version number of the Java tool used and to specify which transcriptomic data was employed as input. This additional information will help others in the field to accurately reproduce the biomass formulation.

We believe there is only one version of the Java tool available, and the version number is not provided by the developers [PMID: 28187415]. As input files, we used protein, DNA, and RNA sequences in FASTA format. We did not use transcriptomic data with this tool. As requested, we have added further details in the text for clarification (lines 203-204).

**REVIEWER #2**

The authors have resolved many technical issues and improved the overall quality of the manuscript.

Minor point:

“It seems that nutrient supplementation alone is not sufficient to sustain growth. Instead, the organism must be able to maintain intracellular energetic and redox balance, for example. Therefore, to address this, we used metabolic modelling as an alternative approach (lines 94-100).”.

Is there a reference for this statement? The growth issue is unclear. There is a distinction between short term limited growth, which several groups have achieved (see Cushion et al. ref # 19) and long-term growth with passages, which has been impossible to achieve so far. This point should be clearly explained in the introduction.

We have now clarified this in the text, as requested (lines 94-99).

**REVIEWER #3**

I thank the authors for their serious effort to provide additional information and to address the reviewers' comments.

I find the manuscript overall strengthened.

My main concerns around predictions made with FBA were already addressed by modifying the ILP problem as a quadratic optimization problem. It is my humble opinion that this misunderstanding can easily happen to others in the community, as the main text and figure 4 describe FBA and its steady state assumption without any adaptation, which led to the points I raised. The authors sufficiently improved the description in the methods section to make this clear.

I kindly request the authors to clarify and strengthen their description also in the "Flux Balance Analysis" section of the main text and Figure 4 (which still describes classical FBA). For instance the authors write "FBA assumes that the metabolic system is in a quasi-steady state, where metabolite production and consumption are balanced within the cell." This was misleading and strictly speaking this is not true, as FBA on its own assumes a steady-state of all systems components. In the current work this was adapted to a quasi-steady state and relaxed bounds requirements (as for example termed "relaxed flux balance analysis" by Fleming et al 2023 in reference 105) to allow for feasible model simulation and subsequent simulation analyses. Excuse me if this may appear nitpicking, I believe the differentiation is important and helps the manuscript if clarified.

In any case, the confusion with strict FBA (and its Sv=0 requirements for all model components) can be easily avoided, if the authors reformulate relevant parts in the Flux Balance Analysis section and update Figure 4 to clarify that they used an adapted FBA approach to relax bounds with a quasi-steady state as detailed out in the methods section.

To clarify this point, we have made the recommended changes in the "*Flux Balance Analysis*" section (lines 224-225, 246-250, 259-265) as well as updated the legend to Figure 4 (lines 819-821), as requested. In addition, to clarify this further, we now highlight that we used relaxed, non-classical FBA in the Abstract (line 30) and Author Summary (line 46).

**DATA AVAILABILITY**

Have the authors made all data and (if applicable) computational code underlying the findings in their manuscript fully available?

Yes, we have included a Data availability statement in the text (lines 855-864).

**Reviewer #1**: No: The "DATA AVAILABILITY" section mentions github repositories of software tools that the authors have used for their analysis. The code, that the authors used for their own analysis (metabolic network reconstruction and anaylsis), is not provided.

We believe this is a misunderstanding. We included the following statement in the text (lines 856-864).

“The code used for the model’s reconstruction and FBA can be found in the following GitHub repository (version 0.6.3 was used in this study, tag 6.3 in the code): <https://github.com/Constellab/gws_gena>

The code used for the RL approach can be found in the following GitHub repository:

<https://github.com/nevolga/optmed>

Note that a digital lab is to be created either on premises or in a cloud to run the code. The guidance can be found here:

<https://constellab.community/bricks/gws_academy/latest/doc/getting-started/b38e4929-2e4f-469c-b47b-f9921a3d4c74>”

**Reviewer #2**: Yes

**Reviewer #3**: None
