# Supplementary material for: Metabolic modelling as a powerful tool to identify critical components of Pneumocystis growth medium
Source: PLoS Comput Biol. 2024 Oct 28;20(10):e1012545. doi: 10.1371/journal.pcbi.1012545 (PMC11542897; doi:10.1371/journal.pcbi.1012545)
Supplement: Attachment — Submitted filename: Response to Reviewer Comments.docx [file pcbi.1012545.s012.docx]

**RESPONSE TO REVIEWS – PCOMPBIOL-D-24-00648R1**

We are grateful to the Reviewers for the additional comments and suggestions. Our responses are detailed below.

**Reviewer #1**

I appreciate the detailed clarification. With the modifications in the manuscript, it is now more clearly illustrated that the presented results are specific to the assumption that fluxes are not constrained by strict steady-state conditions.

Regarding the authors' response, they stated: "we do not say that Pneumocystis is auxotrophic for all 20 amino acids." However, the manuscript contains the following sentence: "[...] Pneumocystis has partially lost the gluconeogenesis pathway and glyoxylate cycle and is incapable of synthesizing all 20 essential amino acids de novo [...]". These statements appear to be contradictory.

Previously we attempted to address this concern by pointing out why these statements are not contradictory. [Previously we said that *Pneumocystis* has lost the capacity to synthesise amino acids from scratch (i.e. *de novo*) but that the fungus does seem able to interconvert certain amino acids (meaning that it does not depend on one of these amino acids for growth if the other is present). Hence, formally, *Pneumocystis* is likely to be auxotrophic for some, but not all amino acids.] However, it would appear that some confusion remains. Therefore, we have attempted to make this even clearer in the text by: (a) changing “*de novo”* to “from scratch*”* on line 173; and (b) adding a sentence to clarify that “auxotrophy for certain specific amino acids may be circumvented via supplementation by others” on lines 180-181.

I understand that, due to the quasi-steady state assumption (QSSA) approach, auxotrophies cannot be predicted based solely on zero flux values for biomass formation when a specific amino acid is removed. However, given that the aim of the study is to contribute to the identification of a medium that supports Pneumocystis growth, readers might reasonably expect a discussion on the results in relation to auxotrophies. In the current version of the manuscript, the focus is on the contribution of amino acids to biomass production, which does not provide new insights regarding potential auxotrophies. If the chosen approach (QSSA-FBA) does not allow for auxotrophy prediction, it would be helpful to clearly state this as a limitation in the discussion.

We have now added this in the Discussion, as requested (lines 509-514).

**Reviewer #2**

The authors have adressed most of the concerns.

**Reviewer #3**

My remaining comments have been addressed by the authors. The differentiation between relaxed FBA and classic FBA is sufficiently clear now.

**DATA AVAILABILITY**

Have the authors made all data and (if applicable) computational code underlying the findings in their manuscript fully available?

Yes, we have included a Data availability statement in the text (lines 864-866).

**Reviewer #1**

Yes

**Reviewer #2**

Yes

**Reviewer #3**

No: The authors provide the reconstructed SBML model as supplementary file.

Provided links refer to the proprietary software workbench Constellab and the description of generating a workflow.

However, custom analysis code that led to the presented results are not available.

The mentioned "digital lab" description should be made available.

We believe this is a misunderstanding. The following Data Availability Statement is included in the text (lines 865-866):

“All the necessary files and instructions to reproduce the results from the paper can be found in the following GitHub repository: <https://github.com/nevolga/optmed>”.

To clarify, we have updated the README file in the repository to make it clear that the digital lab, which is required to run the code and reproduce the results, can be installed and used as open-source software. We have provided detailed installation instructions, along with all the necessary files and steps needed to reproduce the results from the study. Everything required for reproducing the analysis, including the custom workflows, is now freely available in the repository.
